# MICA-G129R: A bifunctional fusion protein increases PRLR-positive breast cancer cell death in co-culture with natural killer cells

**DOI:** 10.1371/journal.pone.0252662

**Published:** 2021-06-02

**Authors:** Hui Ding, Garrett W. Buzzard, Sisi Huang, Michael G. Sehorn, R. Kenneth Marcus, Yanzhang Wei

**Affiliations:** 1 Department of Biological Sciences, Clemson University, Clemson, South Carolina, United States of America; 2 Department of Genetics and Biochemistry, Clemson University, Clemson, South Carolina, United States of America; 3 Department of Chemistry, Clemson University, Clemson, South Carolina, United States of America; Duke University School of Medicine, UNITED STATES

## Abstract

Breast cancer cells were reported to up-regulate human prolactin receptor (PRLR) to assist their growth through the utilization of prolactin (PRL) as the growth factor, which makes PRLR a potential therapeutic target for breast cancer. On the other hand, advanced cancer cells tend to down-regulate or shed off stress signal proteins to evade immune surveillance and elimination. In this report, we created a fusion protein consisting of the extracellular domain of MHC class I chain-related protein (MICA), a stress signal protein and ligand of the activating receptor NKG2D of natural killer (NK) cells, and G129R, an antagonistic variant of PRL. We hypothesize that the MICA portion of the fusion protein binds to NKG2D to activate NK cells and the G129R portion binds to PRLR on breast cancer cells, so that the activated NK cells will kill the PRLR-positive breast cancer cells. We demonstrated that the MICA-G129R fusion protein not only binds to human natural killer NK-92 cells and PRLR-positive human breast cancer T-47D cells, but also promotes NK cells to release granzyme B and IFN-γ and enhances the cytotoxicity of NK cells specifically on PRLR-positive cells. The fusion protein, therefore, represents a new approach for the development of breast cancer specific immunotherapy.

## Introduction

Breast cancer is the most common diagnosed and cause-of-death cancer in women all over the world [1]. Most breast cancer targeting therapies aim at the three receptors: estrogen receptor (ER), progesterone receptor (PR), or epidermal growth factor receptor 2 (HER2). ER and PR are hormone receptors. Tumors expressing ER or PR can be targeted though hormone therapy, either using hormone antagonists to block the receptors, e.g. tamoxifen [2], or inhibitors to block the production of the hormones, e.g. anastrozole or letrozole [3]. HER2 is a breast cancer biomarker. Its overexpression associated with the increased disease recurrence and a poor prognosis of certain aggressive types of breast cancer and can be targeted by antibodies or HER2 inhibitors, e.g. trastuzumab, pertuzumab and lapatinib [4]. However, breast cancer is a heterogeneous disease. Fifteen to 20% of breast cancers do not express these receptors. They are classified as triple negative breast cancer which is the most lethal subtype of breast cancer because of its high heterogeneity, high metastasis frequency, early relapse after standard chemotherapy, and lack of efficient treatment options [5,6]. Finding new target is an urgent need to broaden the therapeutic spectrum in breast cancer. Prolactin (PRL) produced primarily by the anterior pituitary is a polypeptide hormone promoting mammary gland development and milk production [7]. Many studies linked PRL to the pathogenesis and invasion of breast and gynecologic cancers [8–10]. Prolactin receptor (PRLR), structurally homologous with growth hormone receptor, was reported to have a high level of expression in more than 80% of human breast cancer cells and tissues, and contribute to the development of breast cancer [8,11–13], which implicates PRLR could be used as a potential target for breast cancer treatment. G129R is a variant of PRL with a single amino acid substitution mutation which converts it to a PRL antagonist. Instead of sending promoting signals, binding of G129R to PRLR blocks the signal transduction and induces apoptosis in breast cancer cells, and prolonged treatment with G129R induces the accumulation of redundant autolysosomes in 3D cancer spheroids, resulting in autophagy-related cell death [14,15].

Natural killer (NK) cells are a subset of innate cytotoxic lymphocytes serving a critical role in tumor immunosurveillance. NK cells integrate the signals from its activating and inhibitory receptors to target malignant and virus infected cells [16]. The natural killer group 2, member D (NKG2D) receptor is a major type of activating receptors on NK cells and functions in both innate and adaptive immunities [17,18]. When binding to its ligands, NKG2D triggers activation of NK cells to secrete cytokines (e.g. IFN-γ) to recruit and activate other immune cells and release the contents in the cytotoxic granules of NK cells (e.g. granzymes and perforin) to directly kill target cells [19]. Perforin forms pores on the membrane of the target cells. Granzymes diffuse through the pores into the target cells and trigger reactive oxygen species (ROS) mediated cell death. To block granzymes can suppress the cytotoxicity mediated cell death [20–22]. Ligands of NKG2D are a group of stress-induced proteins expressed on virally infected or malignant cells and rarely expressed on healthy cells [23]. They are all homologous to major histocompatibility complex (MHC) class I proteins and belong to two families in humans: MHC class I chain-related proteins (MIC, e.g. MICA, MICB) and UL16-binding proteins (ULBP, e.g. ULBP1-6) [24,25]. Cells expressing these ligands will be detected by NK cells and eliminated by the immune system. However, advanced tumor cells tend to down-regulate or shed off NKG2D ligands to evade immune elimination [26].

Many strategies have been developed to target NKG2D receptor or NKG2D ligands in cancer immunotherapy, including up-regulation of NKG2D or NKG2D ligands, grafting NKG2D or NKG2D ligands with antibodies, cytokines, death receptor or signaling domains of activating receptors [27]. For example, MICA has been overexpressed [28], fused with antibodies [29–32] and cytokines [33] in cancer therapeutic studies.

In this study, we created a novel fusion protein consisting the extracellular domain of MICA and the PRL variant G129R. We hypothesized that the fusion protein MICA-G129R will bridge NK cells and PRLR-positive breast cancer cells. When binding to PRLR, the fusion protein labels the breast cancer cells with MICA, which attracts and activates NK cells to kill the breast cancer cells.

## Materials and methods

### Construction of vectors

The MICA extracellular domain sequence (c.-23 to c.894) was amplified by PCR from a pcDNA3.1_MICA/IL-12 vector constructed in our lab [33] with the 5’ primer CCCaagcttGAGAGGGTGGCGACGTCGGGG and the 3’ primer TCTggatccAGAACCACCACCAGAACCACCACCAGAACCACCCCCAGAGGGCACAGGGTG that contained a (GGGS)×3 linker and cloned into vector pcDNA3.1/Zeo(+) (ThermoFisher Scientific, V86020). The G129R sequence (c.85 to c.681) without the signal peptide (p.1-28) and stop codon, was amplified by PCR from a pCR3.1_G129R plasmid provided by Dr. Wen Y. Chen as a gift with 5’ primer CCggatccTTGCCCATCTGTCCCGG and the 3’ primer CGCctcgagGCAGTTGTTGTTGTGGATGATT and cloned into vector pLenti7.3 (ThermoFisher Scientific, K5325-20). Then the sequences of G129R with the V5 tag and the 6×His tag in the pLenti7.3 vector were amplified by PCR with the 5’ primer CCggatccTTGCCCATCTGTCCCGG and the 3’ primer TGAgcggccgcTCAATGATGATGATGATGATGACCGGTACGCGTAGAATC containing a stop codon in the 3’ primer and cloned into the vector pcDNA3.1/Zeo(+) at the 3’ end of MICA. The sequence of the fusion gene MICA-G129R with the V5-tag and 6×His tag was confirmed by DNA sequencing.

To make control vectors, MICA extracellular domain (c.-23 to c.894) was cloned into the pcDNA3.1/Zeo(+) vector using 5’ primer CCCaagcttGAGAGGGTGGCGACGTCGGGG and the 3’ primer TGAgcggccgcTCACCCAGAGGGCACAGGGTG containing a stop codon; G129R sequence (c.1 to c.684) with signal peptide (p.1-28) and stop codon was directly cut from the pCR3.1_G129R plasmid and inserted into pcDNA3.1/Zeo(+) vector. To make a PRLR expression vector, the full length cDNA sequence of PRLR long isoform was amplified by PCR from a PRLR cDNA plasmid (Sino Biological, HG10278-UT) with 5’ primer tggCTTAAGccaccATGGAGGAAAATGTGGCATCTGC and 3’ primer AAGActcgagTCAGTGAAAGGAGTGTGTAAAACATGC, and cloned into pcDNA3.1(+) (ThermoFisher Scientific, V790-20). All these inserted sequences in the vectors were confirmed with DNA sequencing.

### Cell culture

T-47D, NK-92, 293 and HeLa cell lines were obtained from the American Type Culture Collection (ATCC, Manassas, VA, USA) and cultured following the methods recommended by ATCC at 37°C in a 5% CO_2_ humidified incubator. All the media, fetal bovine serum and donor horse serum were purchased from Corning (NY, USA); other supplements were from ThermoFisher Scientific (MA, USA). Two hundred units per ml of recombinant human IL-2 from PeproTech (NJ, USA, Cat. 200–02) was used in NK-92 culture.

### Transfection and establishment of stable clone

All the cell transfections were carried out using Lipofectamine 2000 (ThermoFisher Scientific) following the instruction of the manufacturer. To obtain stable clones, the transfected cells were cultured in selecting media containing 400 μg/ml of zeocin (InvivoGen, ant-zn-5) for pcDNA3.1/Zeo(+) vector transfection or 500 μg/ml of Geneticin (ThermoFisher Scientific, 10131035) for pcDNA3.1(+) vector transfection. After selection, 3 to 5 cell clones in each transfection were picked and respectively cultured. The protein expression of the transfected gene was verified with Western blot for each clone. The clone expressing the most protein of the transfected gene was used in the study.

### Conditioned media

To test the production of MICA-G129R fusion protein by the transfected cells, 4 × 10^6^ cells of 293/MICA-G129R stable clone were cultured in a T75 flask with 12 ml of culture media without zeocin. A 50 μL sample of the conditioned media was collected from the culture every 24 hours from day 1 to day 5. The media were centrifuged at 1000 g for 15 minutes, then 40 μl of supernatant was collected and froze at -80°C until examination. Forty microliters of fresh medium were used as the conditioned medium of day 0. For preparation of conditioned media, 4 × 10^6^ of 293 cells without transfection (for control conditioned media), 293/MICA-G129R cells (for MICA-G129R conditioned media), 293/MICA cells (for MICA conditioned media) or 293/G129R cells (for G129R conditioned media) were respectively cultured in 12 ml culture media without zeocin for 72 hours. The supernatants were collected, centrifuged at 1000 g for 15 minutes, passed through 0.22 μm filters and stored at -80°C until use.

### Western blot

The conditioned media or the conditioned media diluted with fresh completed media to the desired concentrations were mixed with protease inhibitor cocktail (Cell Signaling Technology, #5871) with a final concentration of 1% and loading buffer (10% SDS, 500mM DTT, 50% Glycerol, 250mM Tris-HCL and 0.5% bromophenol blue dye, PH6.8) with a final concentration of 20%, and then boiled for 10 minutes. All the prepared loading samples was loaded to the gel with the same volume if not specified. The proteins in the samples were separated by SDS-PAGE (10% or 12% polyacrylamide gels) and then transferred onto 0.45 μm pore-size nitrocellulose membranes (Bio-Rad). The proteins on the membrane were stained with ponceau S to confirm that the samples were loaded equally across all lanes. The membranes were then blocked with 5% non-fat dry milk in TBST (10 mM Tris, pH 8.0, 150 mM NaCl, 0.05% Tween-20) overnight. The proteins were detected with primary antibodies followed by secondary antibodies conjugated with horseradish peroxidase (HRP). Blots were developed using enhanced chemiluminescence (ECL) detection reagents (ThermoFisher Scientific, 32209) and exposed to X‑ray films. The films were scanned and quantified using ImageJ (National Institutes of Health). The following antibodies were used in Western blot: monoclonal mouse anti-human MICA (sc-137242), PRL (sc-46698), PRLR (sc-377098) antibodies, mouse IgG light chain binding protein conjugated to HRP (mIgG BP-HRP) (sc-516102) as secondary antibody (purchased from Santa Cruz Biotechnology, CA, USA) and monoclonal mouse anti-V5 antibody (#80076) (purchased from Cell Signaling Technology, MA, USA).

### Immunofluorescent staining

Two hundred thousand T-47D cells were seeded on two glass coverslips in two 35 mm dishes with culture media and cultured overnight. After the media were removed, one coverslip was covered with control conditioned media, and the other one was covered with MICA-G129R conditioned media. After incubation for two hours, the slides were washed with PBS, fixed with 4% formaldehyde, blocked with 1% BSA for 30 minutes, incubated with mouse anti-human MICA (Santa Cruz Biotechnology, sc-137242) primary antibody for one hour, and then chicken anti-mouse IgG secondary antibody conjugated with Alexa Fluor 488 (ThermoFisher Scientific, A-21200) for one hour. The slides were mounted using mounting buffer with DAPI and observed with a fluorescence microscope (LEICA DMi8).

### Flow cytometry

One million NK-92 cells were collected and equally separated into two groups. They were respectively incubated with control conditioned media or MICA-G129R conditioned media for two hours. The cells were then harvested, washed with PBS on ice, blocked with 1% BSA for 10 minutes, incubated with mouse anti-human PRL (Santa Cruz Biotechnology, sc-46698) primary antibody for 20 minutes, and incubated with chicken anti-mouse IgG secondary antibody conjugated with Alexa Fluor 488 (ThermoFisher Scientific, A-21200) for 15 minutes. After three times PBS wash, the cells were pelleted and resuspended in PBS, and then analyzed using flow cytometer (BD Accuri C6).

### Cytotoxicity assay

The target cells including T-47D cells, HeLa cells, 293 cells, 293/PRLR cells were seeded in 96-well plates and incubated overnight. Then the media was removed and replaced with 50% Alpha Minimum Essential Medium and 50% of conditioned media with NK-92 cells at the effector/target ratios of 5:1, 2:1, 1:1 and 1:2. The co-cultures were incubated in a 37°C, 5% CO_2_ humidified incubator for 24 hours. After centrifugation at 500 g for 5 min, 50 μl of supernatant was collected from each well for determining the cytotoxicity using the CytoTox 96 nonradioactive cytotoxicity assay (Promega, G1780) following the manufacturer’s protocol. The assay determines the cytotoxicity by measuring the lactate dehydrogenase (LDH), a stable cytosolic enzyme, released by the lysed cells. For each conditioned media treatment, many control wells were set at the same time for the calculation of the cytotoxicity: NK cells only (for effector cell spontaneous LDH release), target cells only (target cell spontaneous LDH release), target cells with lysis solution (for target cell maximum LDH release) and medium only (background).

### MICA-G129R fusion protein purification

Fusion protein MICA-G129R in supernatant of 293/MICA-129R stable clone was purified from the conditioned media using nickel sepharose beads (Ni Sepharose™ 6 Fast Flow, GE Healthcare, 17-5318-02). Five hundred microliters of beads were mixed with 90 ml MICA-G129R conditioned media and rotated overnight at 4°C. The slurry was applied to a column to allow the beads to pack. The MICA-G129R conditioned media was collected after passing through the column twice. Then the column was washed with 10 ml high salt Buffer A (20 mM K_2_HPO_4_ pH 7.5, 10% glycerol, 0.5 mM EDTA, 0.01% IGEPAL and 1 mM DTT) containing 1 M KCl and 25 mM imidazole followed by a wash with 10 ml low salt Buffer A containing 150 mM KCl and 25 mM imidazole. The bound MICA-G129R protein was eluted by adding 0.5 ml of Buffer A containing 150 mM KCl and 500 mM imidazole to the beads, incubated for 5 min, then the column was drained and the elution buffer containing MICA-G129R protein was collected. This elution step was repeated for two more times. The three elution fractions containing the purified MICA-G129R were pooled, passed through a 0.22 μm filter and stored at -80°C until use.

### High-performance liquid chromatography (HPLC)

The MICA-G129R conditioned media and the purified MICA-G129R protein solution were diluted with PBS at 1:20 and 1:5 ratios, and then analyzed using reversed-phase HPLC. Polypropylene trilobal capillary-channeled polymer (C‐CP) fibers were packed as the HPLC column as previously described [34]. The separation procedures were carried out following a method built previously [35].

### Protein quantification

The proteins in the purified MICA-G129R solution were quantified using Pierce BCA Protein Assay (ThermoFisher Scientific, 23227) according to the manufacture’s introduction. The provided albumin (BSA) in the assay was made serial dilutions as the protein standards.

### ELISA

To examine the release of granzyme B and IFN-γ of NK-92 cells, NK-92 cells were cultured with or without T-47D cells at the ratio of 1:1 in the presence of the purified MICA-G129R protein at a final concentration of 125 nM (equivalent to the MICA-G129R concentration in the mixed media with 50% MICA-G129R conditioned media used in the cytotoxicity assays) or the elution buffer in the protein purification as control. After 6 hours incubation and a quick spin, the supernatant was collected for ELISA. Human granzyme B ELISA kit (B&D Systems, DY2906-05) and Human IFN-γ uncoated ELISA kit (Invitrogen, 88–7316) were used for the ELISA measurements according to the manufacture’s protocols.

### Caspase-3 activity measurement

T-47D cells alone or with NK-92 cells at the ratio of 1:1 were cultured with the purified MICA-G129R protein at the final concentration of 125 nM or the elution buffer from the protein purification as control for 6 hours. After removal of the media or media with the floating NK-92 cells, the T-47D cells of each treatment were washed with PBS and lysed. The total proteins in the cell lysate of each sample were measured using BCA assay. The same amount of total proteins from each sample was used for detecting the caspase-3 activity using the Capspase-3 fluorometric kit (R&D Systems, BF1100) according to the manufacture’s instruction.

### Statistical analysis

In all the experiments, each treatment was performed in three replicates unless otherwise indicated and the experiments were repeated at least twice. The results were expressed as the mean ± standard deviation (SD). The significant differences were evaluated using either two-tailed Student’s *t*-test (for comparisons between two groups) or one-way analysis of variance (ANOVA) (for comparisons between three or more groups) and then Tukey’s HSD test. P < 0.05 was considered to indicate a statistically significant difference.

## Results

### Construction of MICA-G129R fusion gene in expression vector

**Fig 1:**
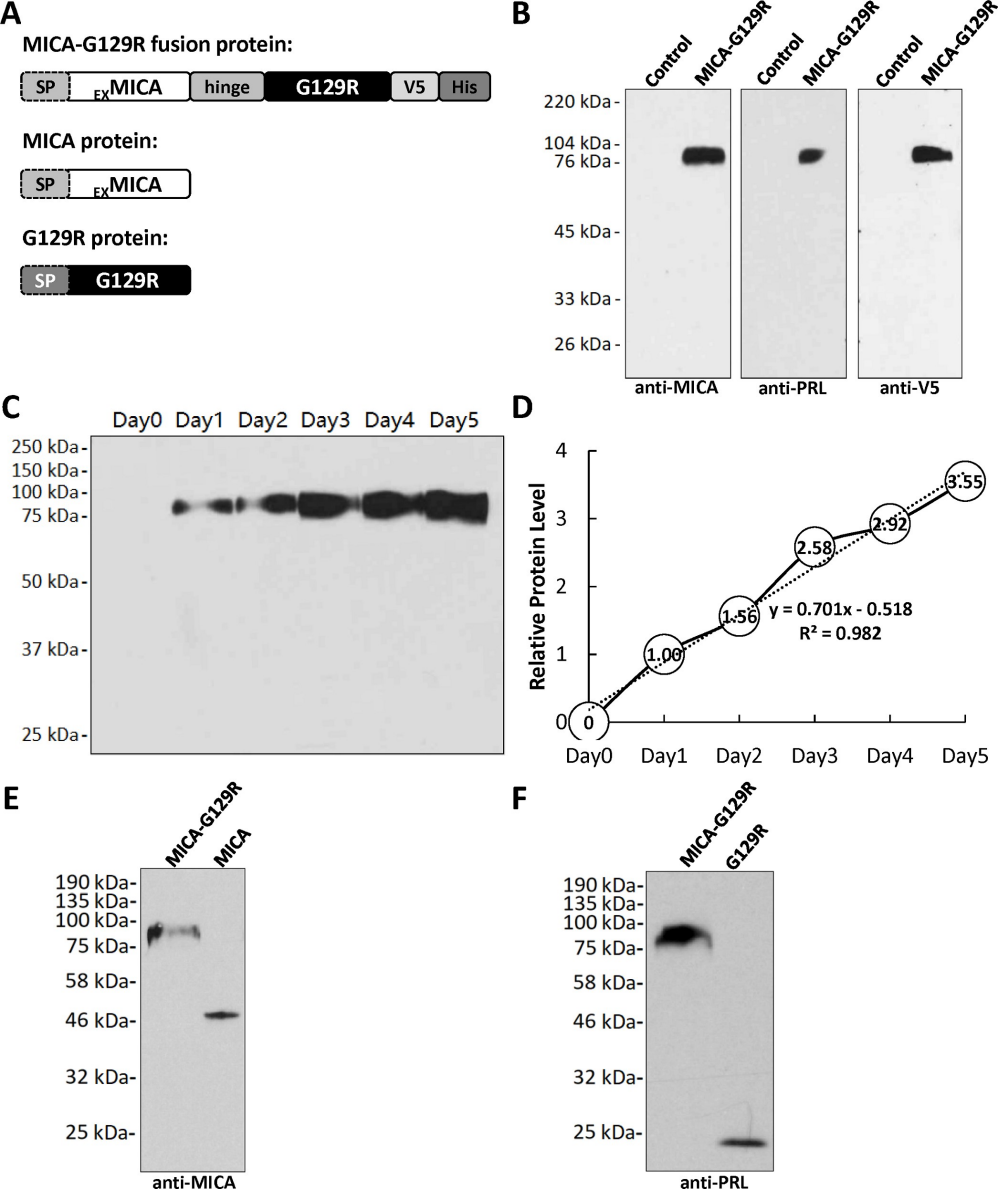
Expression of MICA-G129R fusion protein. **A.** Gene constructs of the MICA-G129R fusion protein, MICA protein and G129R protein. The gene sequences encoding the extracellular domain of MICA and G129R (without signal peptide), were cloned, linked together with a (GGGS)×3 hinge and inserted into the expression vector. A V5 tag and a His tag were added to the 3’ end of the MICA-G129R sequence. The gene sequences of the extracellular domain of MICA or the G129R (with signal sequence) were also respectively cloned into the expression vectors as controls. The vectors were transfected into 293 cells, and the highest productive stable clone was selected. **B.** The presence of MICA-G129R fusion protein in the conditioned media was confirmed using anti-MICA, anti-PRL and anti-V5 antibodies. The same volume of conditioned media collected from the untransfected 293 cells was used as the control. **C.** The MICA-G129R protein in the conditioned media was detected using anti-MICA antibody in Western blot from day 0 to day 5. The loading volumes of the conditioned media from each day were the same and well controlled. **D.** The qualification of the Western blot in (**C**). All the MICA-G129R protein levels were normalized to the MICA-G129R protein level of day 1. The MICA expression vector and G129R expression vector were also transfected into 293 cells and the stable clones were selected. **E** and **F.** The MICA protein and the G129R protein in the respective conditioned media were detected using Western blot beside the MICA-G129R conditioned media. SP, signal peptide.

The gene sequences encoding the extracellular domain of MICA and the PRL mutant, G129R were cloned and inserted into the mammalian expression vector pcDNA3.1/Zeo(+). A (GGGS)×3 linker sequence was added to hinge the MICA and G129R and provide flexibility. A V5 tag and a His tag were aligned downstream of the MICA and G129R segments in the open reading frame in the vector (Fig 1A). The gene sequences encoding the extracellular domain of MICA or G129R were also cloned respectively into the vector pcDNA3.1/Zeo(+) as the MICA vector or G129R vector (Fig 1A). All the insertions and junctions within the vectors were confirmed by DNA sequencing. There are signal peptides at the N-terminus of both the MICA and G129R which lead the MICA and G129R to be secreted outside of the cells. The signal peptide of MICA was kept for the secretion of fusion protein MICA-G129R and the signal peptide of G129R was removed.

### Expression of MICA-G129R fusion protein

The MICA-G129R expression vector was transfected into 293 cells that do not express detectable MICA or PRL proteins. Clones resistant to the antibiotic zeocin were selected and expanded. The MICA-G129R fusion protein in the conditioned media from the clones was confirmed using anti-MICA, anti-PRL and anti-V5 antibodies (Fig 1B). The clone with the highest MICA-G129R protein expression was chosen to produce the fusion protein for subsequent studies. The accumulation of the MICA-G129R protein in the conditioned media was also investigated (Fig 1C and 1D). We found that fusion protein MICA-G129R was linearly produced and accumulated in the culture media in the 5-day culture. Considering the accumulation of cellular waste and the depletion of the nutrients in the media, we did not conduct a longer culture.

Expression vectors for MICA alone and G129R alone were respectively transfected into 293 cells. Stable clones were established and the expression of the MICA protein or G129R protein was confirmed by Western blot analysis (Fig 1E and 1F).

### MICA-G129R binds to T-47D cells and NK-92 cells

**Fig 2:**
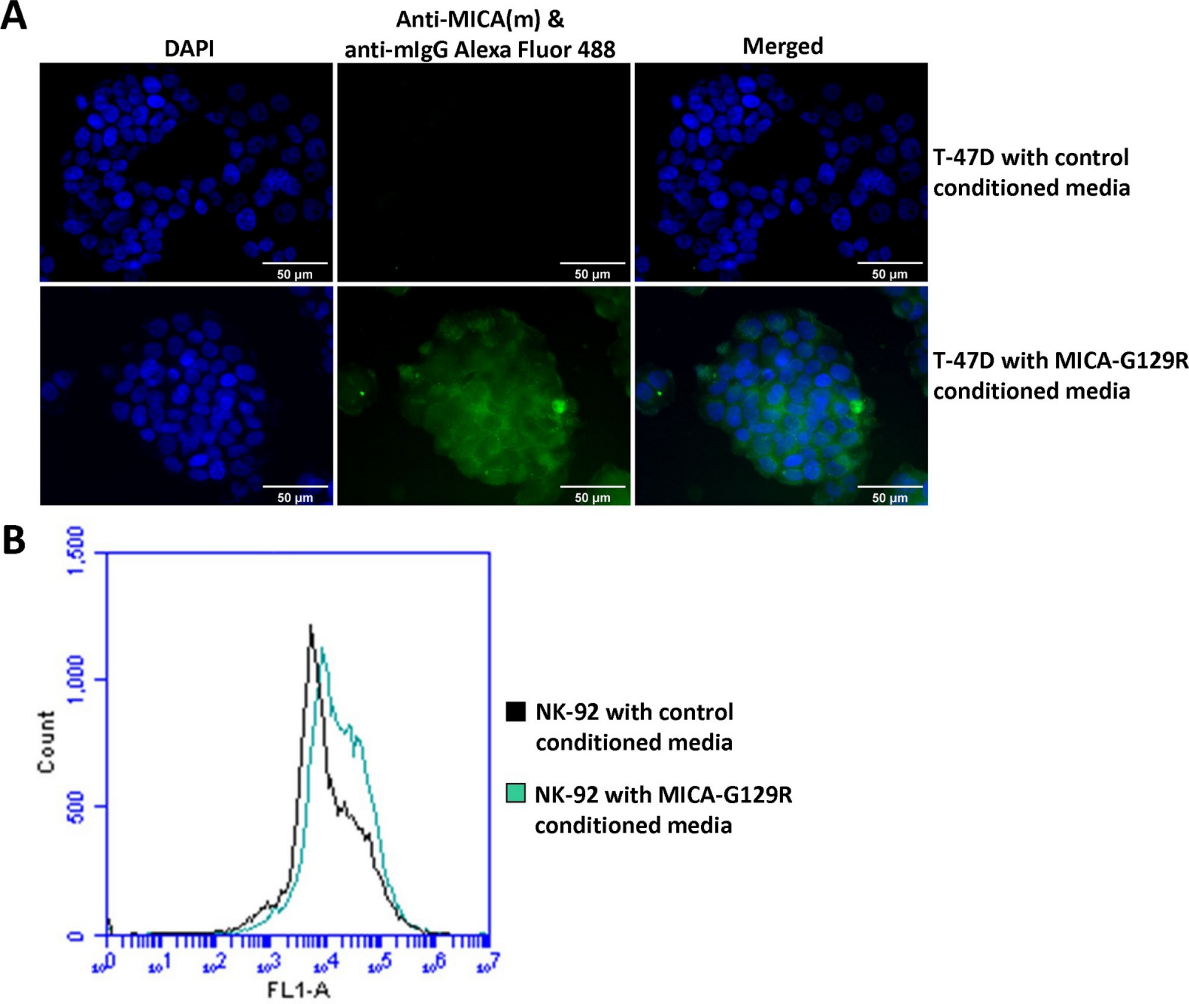
Fusion protein MICA-G129Rbinds to T-47D cells and NK-92 cells. Control or MICA-G129R conditioned media were incubated with (**A**) T-47D cells or (**B**) NK-92 cells for two hours. T-47D cells were fixed and first stained with mouse anti-human MICA primary antibody followed by anti-mouse IgG Alexa Fluor 488 secondary antibody and DAPI, and observed with fluorescence microscope. NK-92 cells without fixation were stained with mouse anti-PRL primary antibody followed by an anti-mouse IgG Alexa Fluor 488 secondary antibody. The stained NK-92 cells were analyzed with flow cytometry. FL1 indicates the 530/30 nm standard interference filter.

To investigate if fusion protein MICA-G129R can bind to breast cancer cells, PRLR-positive breast cancer cell line T-47D cells were incubated with control or MICA-G129R conditioned media and then stained with mouse anti-MICA primary antibody followed by an anti-mouse secondary antibody conjugated with green fluorescent dye Alexa Fluor 488. The T-47D cells incubated with MICA-G129R conditioned media showed strong green fluorescence, while the cells with control conditioned media did not (Fig 2A), demonstrating that the fusion protein MICA-G129R in the conditioned media binds to PRLR-positive breast cancer T-47D cells.

A human NK cell line, NK-92, was also incubated with the control and MICA-G129R conditioned media and stained with mouse anti-PRL primary antibody followed by an anti-mouse secondary antibody conjugated with Alexa Fluor 488. The NK-92 cells were then analyzed by flow cytometry. The result shows that the curve of NK-92 cells incubated with the MICA-G129R conditioned media shifted to the right compared to NK-92 cells incubated with the control conditioned media, demonstrating that the MICA-G129R protein also binds to NK-92 cells (Fig 2B).

### MICA-G129R enhances cytotoxicity of NK-92 cells on PRLR-positive breast cancer cells

**Fig 3:**
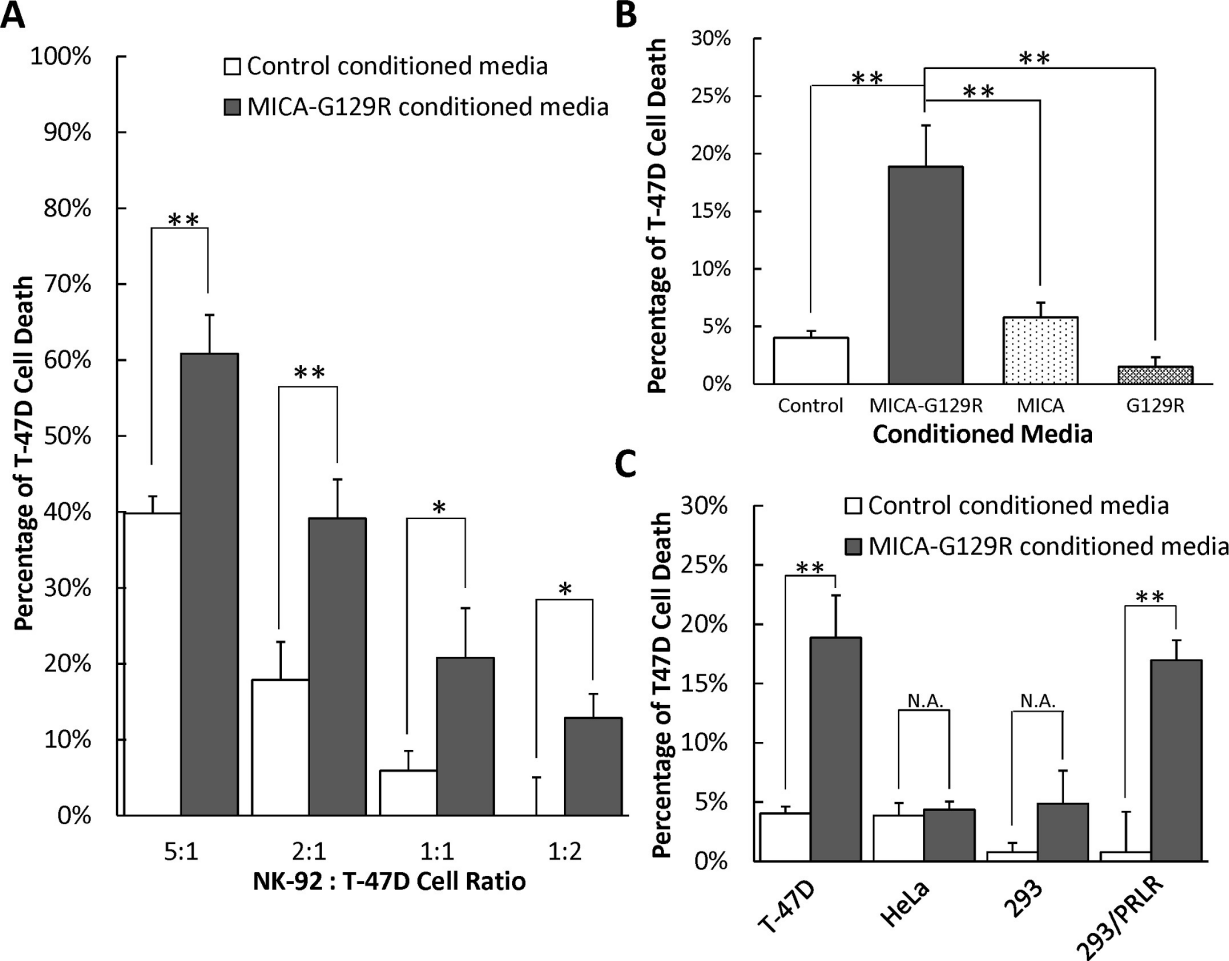
MICA-G129R fusion protein enhances the cytotoxicity of NK-92 cells on PRLR-positive T-47D cells. The cytotoxicity of NK-92 cells on T-47D cells was measured at different conditions. **A.** T-47D cells were co-cultured with NK-92 cells at different effector/target ratios 5:1, 2:1, 1:1, and 1:2 for 24 hours with control or MICA-G129R conditioned media. **B.** The co-culture of T-47D cells and NK-92 cells at the ratio of 1:1 was treated with the control, MICA-G129R, MICA or G129R conditioned media for 24 hours. **C.** The NK-92 cells were co-cultured with T-47D cells, HeLa cells (PRLR-negative), 293 (PRLR-negative) or PRLR ectopic expressed 293 cells at the ratio of 1:1 with control or MICA-G129R conditioned media for 24 hours. Data are presented as mean ± SD (n = 3). * indicates P<0.05; ** indicates P<0.01.

To investigate if fusion protein MICA-G129R could enhance the cytotoxicity of NK cells on PRLR-positive breast cancer cells, the control and MICA-G129R conditioned media were added to the co-culture of NK-92 cells and T-47D cells at different effector/target ratios (5:1, 2:1, 1:1, and 1:2) for 24 hours and the cytotoxicity on T-47D cells was measured using a lactate dehydrogenase (LDH) cytotoxicity assay. Compared to control conditioned media, the MICA-G129R conditioned media induced 21%, 21%, 15% and 13% more T-47D cell death at the different effector/target ratios of 5:1, 2:1, 1:1 and 2:1 (Fig 3A). To confirm that the MICA or G129R alone cannot induce an increased T-47D cell death in the co-culture, MICA conditioned media or G129R conditioned media were applied to the co-culture of NK-92 and T-47D cells. The result revealed that those conditioned media failed to significantly enhance the cytotoxicity of NK-92 cell on T-47D cells (Fig 3B).

To validate that PRLR on the target cells is necessary for fusion protein MICA-G129R’s enhancement of cytotoxicity, PRLR-negative cell lines HeLa cells, 293 cells (S1A Fig), and PRLR long isoform ectopically expressed 293/PRLR cells (S1B Fig) were co-cultured with NK-92 cells in control or MICA-G129R conditioned media. The results indicated that the MICA-G129R conditioned media could not promote the cytotoxicity of NK cells on PRLR-negative HeLa or 293 cells, however the 293/PRLR cells were effectively killed by NK-92 cells in the MICA-G129R conditioned media (Fig 3C).

### Fusion protein MICA-G129R purification

**Fig 4:**
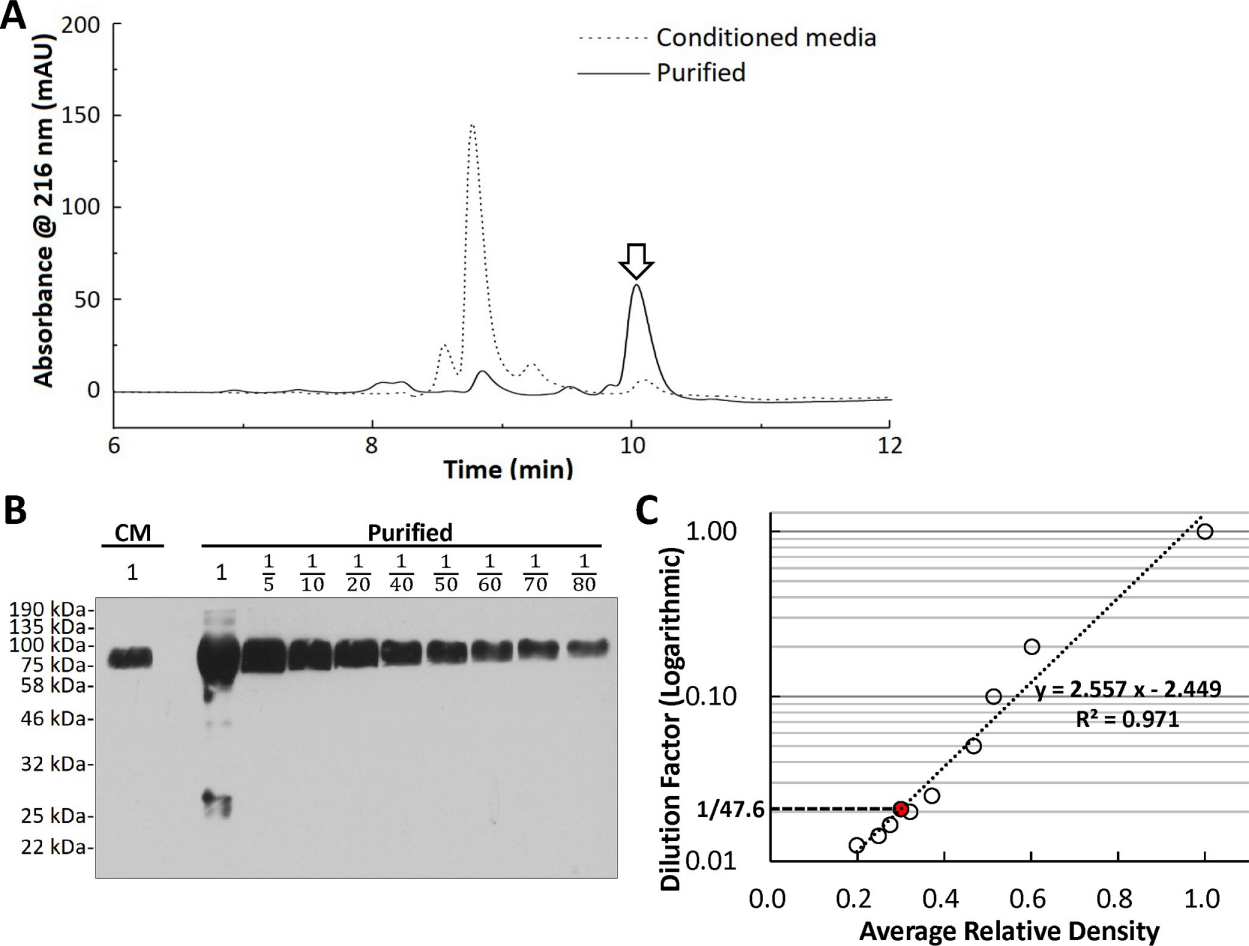
Fusion protein MICA-G129R purification from conditioned media. Nickel resin chromatography was used to purify the fusion protein MICA-G129R from conditioned media. **A.** HPLC analysis of the MICA-G129R conditioned media and the purified MICA-G129R protein solution. The white arrow indicates the peaks of MICA-G129R protein. **B.** Fusion protein MICA-G129R in the conditioned media and serial dilutions of the purified protein were detected using anti-MICA antibody in Western blot. The purified protein was diluted with fresh culture media of 293. CM indicates the lane loaded with the conditioned media before purification. The numbers on the lanes indicate the dilution factors. There is an empty lane between the lane of conditioned media and the lanes of the purified protein dilutions. **C.** Quantification of the Western blot in (**B**). All the bands were quantified based on the average relative densities using ImageJ. The serial dilutions of the purified MICA-G129R protein solution were quantified and modeled by a regression line logarithmically. The red dot shows where the original MICA-G129R conditioned media is on the regression line based on its average relative density.

Fusion protein MICA-G129R was designed with a His tag to assists its purification and concentration. Using the nickel resin chromatography, fusion protein MICA-G129R was successfully purified from the conditioned media. HPLC analysis of the purified MICA-G129R using a C-CP fiber column [34,35] revealed that the peak representing MICA-G129R (indicated with the white arrow in Fig 4A) dramatically increased to 70.0% of the total area under the curve, meaning that the purity of MICA-G129R in the purified solution was 70.0%. The other peaks dampened or disappeared after the purification, which reflected that most of other proteins in the conditioned media were removed. The analysis of the purified MICA-G129R protein using PAGE and Coomassie blue staining (S2 Fig) also showed that the band of MICA-G129R protein was augmented whereas the other bands were diminished or disappeared after purification. The concentration of the total proteins was 1007.5 ± 10.0 μg/ml in the purified solution measured by BCA assay. As 70.0% of the protein was MICA-G129R, the concentration of MICA-G129R protein was 705.3 μg/ml. Based on the amino acid sequence, the molecular weight of fusion protein MICA-G129R (including the V5 tag and His tag, without the signal peptide) was calculated to be 59.2 kDa using an online bioinformatic tool (https://www.bioinformatics.org/sms/prot_mw.html). Therefore, the molar concentration of MICA-G129R protein in the purified solution was 11.9 μM.

To further quantify fusion protein MICA-G129R in the purified solution, the purified solution was serially diluted. The MICA-G129R in the dilutions was detected using Western blot, quantified and analyzed (Fig 4B and 4C). Result shows that fusion protein MICA-G129R was concentrated 47.6 times in the purification. Base on this, it is calculated that the fusion protein MICA-G129R in the conditioned media was approximately 14.8 μg/ml (250.0 nM). As the volume of the MICA-G129R conditioned media was decreased 60-fold in this purification, the recovery rate was 79.3%.

### Fusion protein MICA-G129R activates NK-92 cells

**Fig 5:**
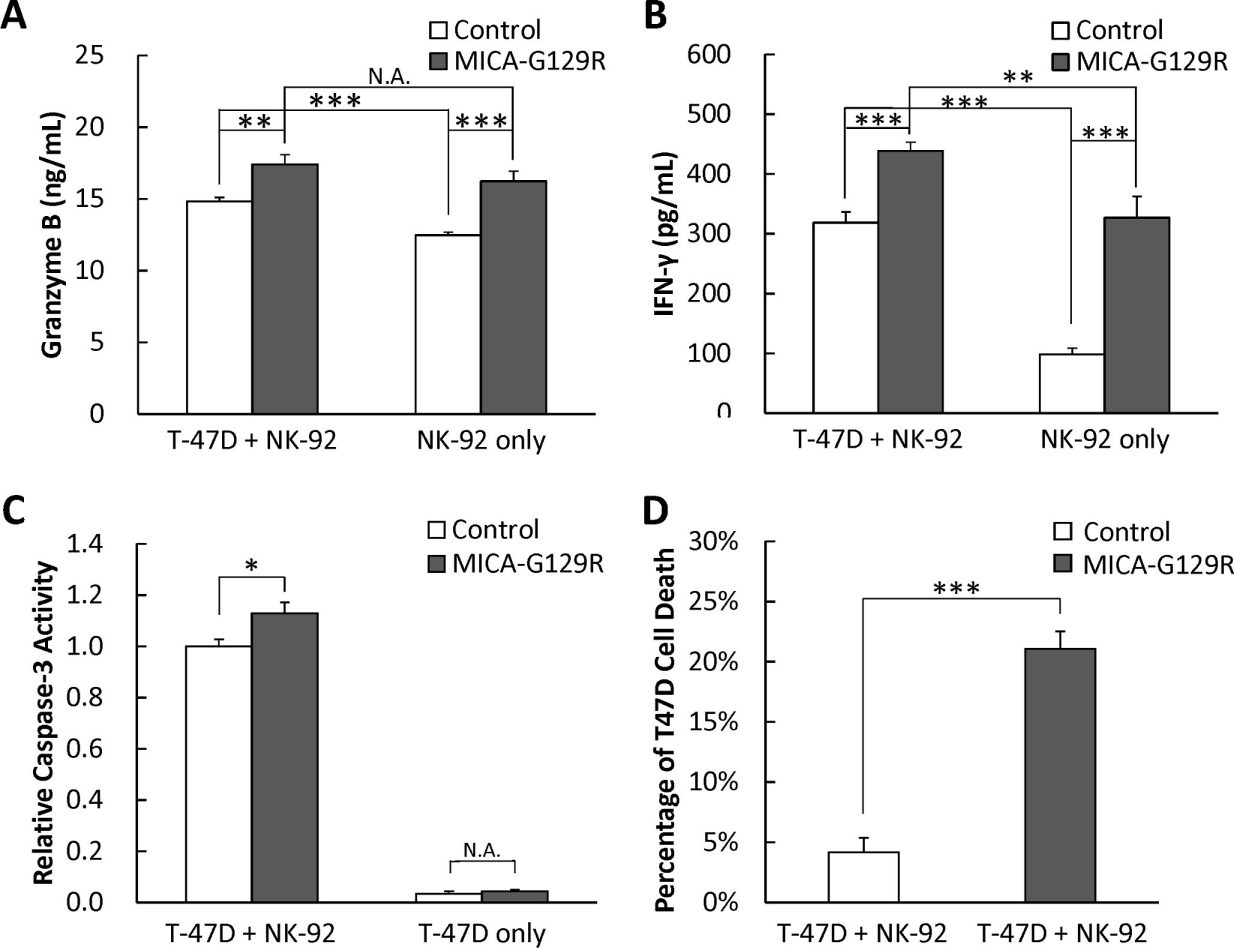
Purified fusion protein MICA-G129R fusion protein activats NK-92 cells. NK-92 cells, T-47D cells, or NK-92 cells with T-47D cells at the ratio of 1:1 were cultured in the media with or without the purified MICA-G129R. After 6 hours, the media were collected for (**A**) ELISA of granzyme B or (**B**) ELISA of IFN-γ. **C.** The activity of caspase-3 in T-47D cells from T-47 cells alone or T-47D cells co-cultured with NK-92 cells was measured using caspase-3 fluorescence assay. **D.** Purified MICA-G129R were evaluated in inducing the cytotoxicity in the co-culture of NK-92 cells and T-47D cells at the ratio of 1:1 for 24 hours. Data are presented as mean ± SD (n = 3). * indicates P<0.05; ** indicates P<0.01; *** indicates P<0.001.

To determine if purified fusion protein MICA-G129R activates NK-92 cells, granzyme B and IFN-γ released by NK-92 were measured in NK-92 alone or NK-92 cells co-cultured with T-47D cells with or without the purified MICA-G129R fusion protein. The elution buffer in the protein purification was used as control. It was found that purified MICA-G129R significantly stimulated NK-92 cells to release granzyme B and IFN-γ in both NK-92 cells alone and co-culture of NK-92 cells with T-47D cells (Fig 5A and 5B). While co-culture with T-47D cells significantly elevated the release of granzyme B and IFN-γ of NK-92 cells, adding MICA-G129R promoted NK-92 cells to release IFN-γ to the level as co-cultured with T-47D cells. MICA-G129R protein also pushed the granzyme B release of NK-92 cells to such a high level that there was no difference between with and without T-47D cells.

To investigate if apoptosis was induced in T-47D cells by the granzyme B and other contents released from the granules of NK cells, the activity of caspase-3 in the T-47D cells was measured using a fluorometric assay after carefully removing the NK-92 cells. The caspase-3 activity in the T-47D cells co-cultured with NK-92 cells and MICA-G129R protein was found significantly higher than that observed in the T-47D cells co-cultured with NK-92 cells but without MICA-G129R. Without NK-92 cells, the activity of caspase-3 in T-47D cells was very low, and MICA-G129R protein could not induce any remarkable difference (Fig 5C).

When the same level of purified MICA-G129R as the conditioned media was added to the co-culture of NK-92 cells and T-47D cells, it induced a significantly increased target cells death (Fig 5D).

## Discussion

Fusion protein MICA-G129R was confirmed not only able to bind to PRLR-positive breast cancer cells and NK cells (Fig 2), but also enhance cytotoxicity of NK cells on PRLR-positive cells (Fig 3A and 3C). When compared with MICA or G129R proteins, the fusion protein MICA-G129R promoted more robust cytotoxicity (Fig 3B) suggesting MICA-G129R may help in bridging the effector cells and target cells. In addition, by comparing T-47D cells with PRLR-negative HeLa cells, and 293/PRLR cells with 293 cells in the co-culture with NK-92 cells, PRLR on the target cells was demonstrated necessary for MICA-G129R to induce the killing from NK cells (Fig 3C). Granzyme B and IFN-γ are two of the major effector proteins in the cytotoxicity procedure of NK cells. Granzyme B released from the granules of NK cells enters target cell via pores formed by perforins and induces apoptosis of target cells. IFN-γ is secreted by activated NK cells to stimulate and regulate other immune cells, like macrophages and T cells, to destroy the target cells. Granzyme B and IFN-γ were both found to be elevated by the MICA-G129R fusion protein (Fig 5A and 5B). Since granzymes released by NK cells can directly cleave and activate caspase-3 to trigger ROS mediated apoptosis [22], caspase-3 activity in the target cells was measured and found significantly elevated when MICA-G129R presented in the co-culture, which further supported that MICA-G129R enhanced cytotoxicity (Fig 5C).

To quickly confirm the concept that fusion protein MICA-G129R induces cytotoxicity of NK cells on PRLR-positive breast cancer cells, conditioned media containing MICA-G129R were initially used in the study. Fusion protein MICA-G129R was then purified and quantified (Fig 4). Very few multimers and truncated proteins were found in the purified proteins. While the calculated molecular weight was 59.2 kDa, MICA-G129R showed a larger molecular weight in the protein gel, which may be due to protein glycosylation or the denatured protein structure especially it is a fusion of two proteins. The purified protein induced the same level of cytotoxicity in the co-culture as the MICA-G129R conditioned media (Figs 3A and 5D). In the results, the reasons for that MICA-G129R showed a larger molecular weight in protein gels than the calculated molecular weight may be due to protein glycosylation or irregular denatured protein structure of the fusion protein.

Different from most of bispecific proteins that typically use antibodies for targeting, fusion protein MICA-G129R uses the antagonistic G129R to specifically target PRLR-positive cells. The PRL/PRLR pathway has been studied for decades because of its etiological role in breast cancer [36]. While PRL tends to be elevated in the serum of breast cancer patients, breast cancer cells also synthesize PRL locally as an autocrine/paracrine growth factor and overexpress PRLR to utilize PRL to promote their growth [37]. Due to the wide expression of PRLR in almost all types of breast cancers, fusion protein MICA-G129R can be used for many types of breast cancers regardless the common classification with ER, PR, HER2 or triple negative breast cancers. PRLR could be another promising antigen to be targeted in breast cancers, especially the ones lacking effective targets, like triple negative cancers. NK cells were also reported expressing PRLR and PRL could stimulate NK cells’ proliferation and cytotoxicity through IL-2 and IL-15 pathways [38]. The effect of G129R or MICA-G129R on NK cells is an open question and will be considered in future studies.

G129R as an antagonist has the great potential to target the PRL/PRLR pathway since its binding to PRLR does not promote growth of breast cancer cells, instead, induces autophagy-related cell death [15]. The G129R conditioned media did not induce significant cell death in this study (Fig 3B), which may due to the short incubation time. G129R also has been well documented as a single agent, in combination with other agents, such as Trastuzumab (anti-HER2) [39], and as fusion proteins with IL-2 [40], endostatin [41] or exotoxin [42]. In addition, as G129R and MICA are either a variant or a part of the intrinsic protein in the body, fusion protein MICA-G129R is less likely to cause unwanted immune problems compared with antibodies and other fusion proteins.

MICA has been reported to be shed from advanced cancer cells to evade NKG2D-mediated immune detection and elimination. The shedding of MICA results in its release into the circulation. Soluble MICA was found significantly elevated in the sera of patients with leukemia [43,44], colorectal cancer [45], prostate cancer [46], lung cancer, ovarian cancer, prostate cancer, breast cancer [47], pancreatic cancer [48,49], oral squamous cell cancer [50,51] and hepatocellular cancer [52]. Shedding of MICA is the result of proteolytic cleavage in the stalk of the ectodomain [53], which has been removed in our fusion protein design. The soluble MICA may complete with MICA-G129R to bind the NKG2D on NK cells, and was reported to be correlated with the NKG2D down-regulation [54]. Fusion protein MICA-G129R when circulating into breast tumor will adhere the MICA back to the surface of breast tumor cells through the binding of the G129R to PRLR. It becomes immobilized and enriched in breath tumor just like the MICA expressed on cancer cell surface, which will attract and activate NK cells and other effector cells into the tumor. The fusion protein may be affected by soluble MICA or itself may suppress the NKG2D expression when not bound to breast cancer cells. However, other factors, such as blocking TGF-β and applying cytokines like IL-2 or IL-18, can overcome the inhibition to NKG2D expression regardless of the soluble MICA in sera [55,56]. *In vivo* study has to be done to answer these questions.

Since NKG2D is also expressed on NKT cells, CD8^+^ T cells, γδ T cells, and some activated CD4^+^ T cells [57], fusion protein MICA-G129R may also attract and activate these cells to fight against breast cancer. The interaction between MICA-G129R and the other NKG2D-expression cell types will be evaluated in the future *in vivo* study with a particular focus on γδ T cells because of their ability to infiltrate solid tumors [58]. The infiltration of immune cells into solid tumors is always a critical challenge for cancer immunotherapy [59,60]. The activation of MICA-G129R to γδ T cells may benefit the treatment to breast cancer.

Direct cell-cell contact between NK cell and the target cell to form an interface structure, immunological synapse, is required in the cytotoxicity of NK cells [61,62]. The results that fusion protein MICA-G129R binds to NK-92 cells and T-47D cells, and it did not affect the viability of T-47D or NK-92 cells, but when induced into the co-culture of T-47D and NK-92 cells, it induced more the T-47D cell death suggest that MICA-G129R helps the formation of immunological synapse and bridges NK-92 and T-47D cells.

One of the limitations of this study is that T47D was the only cell lines used, so more breast cancer cell lines with different PRLR expression levels will be included in future study. *In vivo* mice models will also be included in the study of MICA-G129R for its circulation, tumor-targeting, NK cell-infiltration, efficacy and safety in inducing cytotoxicity and combination with other therapeutics. The *in vivo* interaction of human NK cells and MICA-G129R can be investigated in immunodeficiency mice xenografted with human PRLR-positive breast cancer cells. MICA also binds to mouse NKG2D and activates mouse NK cells [63], so the mouse intrinsic NK cell may be activated and redirected by the fusion protein. The physiological ratios of NK cells to cancer cells in solid tumors could be as low as 1:35 [64], which is quite different from the ratios in *in vitro* studies. This is a big challenge for all the solid tumor immunotherapies. Maybe combination of MICA-G129R with NK cell transfer is a possible way in the *in vivo* studies.

Heterogeneity of tumor-targeted antigens is one of the hallmarks of cancer and one of the obstacles in cancer immunotherapy, especially in solid tumors. Drug-resistance often develops when only one antigen is targeted. The strategy of fusing with MICA can be extended to become a universal strategy. Different antibodies, like Trastuzumab, Pertuzumab (anti-HER2), Matuzumab (anti-EGFR) can all be respectively fused with MICA and used together as a cocktail, so that multiple antigens in cancer can be targeted at the same time. NK cells can be derived from health donors, umbilical cord blood or induced pluripotent stem cells (iPSCs) and engineered to minimize the antigenicity and expand to be allogenic and off-the-shelf, and used together with the MICA fusion proteins. MICA and NKG2D can be engineered to bind more specifically with higher affinity to avoid the interaction with the soluble MICA and the intrinsic NK cells. This platform is like a “multi-bit screwdriver”. The handle of the screwdriver is the engineered allogenous off-the-shelf NK cell, while the MICA fusion proteins are the bits targeting different “screws” on cancer cells.

In conclusion, a novel fusion protein MICA-G129R was created and demonstrated able to bind to PRLR-positive breast cancer cells and NK cells, promote the release of granzyme B and IFN-γ by NK cells and enhance the cytotoxicity to PRLR-positive breast cancer cells.

## Supporting information

S1 Fig PRLR protein expression in HeLa, T-47D and PRLR ectopically expressed 293 cells. **A.** Western blot of PRLR with cell lysates of HeLa and T-47D cells. **B.** Western blot of PRLR with cell lysates of PRLR transfected 293 cells. The PRLR protein in three stable clones of PRLR transfected 293 cells were detected. The untransfected 293 served as the control. The clone 2 was used for the flowing study as the PRLR ectopically expressed 293 cells.
(TIF)

S2 Fig Coomassie blue stained protein gel with MICA-G129R conditioned media and purified MICA-G129R. The white arrow indicates the bands of MICA-G129R. CM indicates the lane with the MICA-G129R conditioned media. M indicates the lanes with the protein standard marker. P indicates the lanes with the purified MICA-G129R protein solution. The loading volume of each sample was indicated above each line.
(TIF)

S1 Raw images (PDF)
