# Supplementary figures and images for: MICA-G129R: A bifunctional fusion protein increases PRLR-positive breast cancer cell death in co-culture with natural killer cells
Source: PLoS One. 2021 Jun 2;16(6):e0252662. doi: 10.1371/journal.pone.0252662 (PMC8172023; doi:10.1371/journal.pone.0252662)

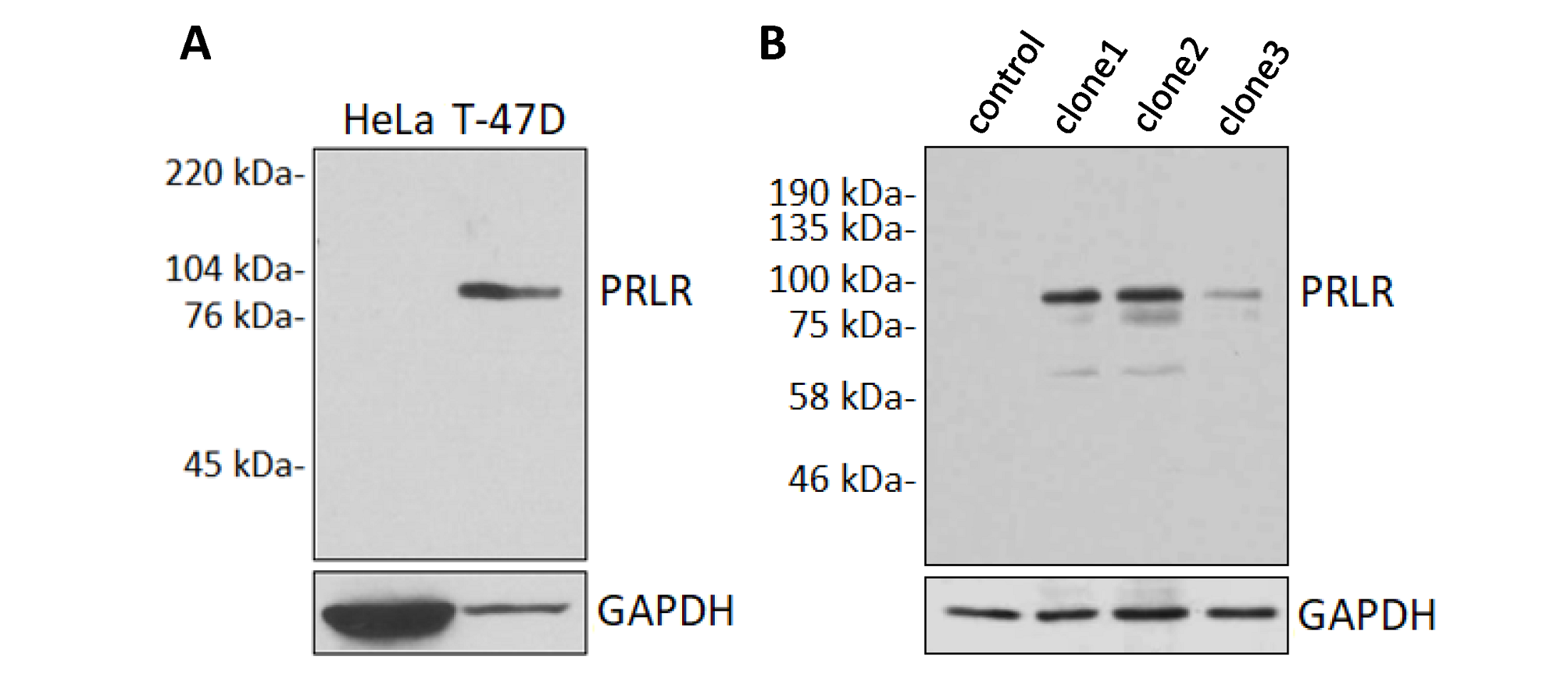

Supplement: S1 Fig — A. Western blot of PRLR with cell lysates of HeLa and T-47D cells. B. Western blot of PRLR with cell lysates of PRLR transfected 293 cells. The PRLR protein in three stable clones of PRLR transfected 293 cells were detected. The untransfected 293 served as the control. The clone 2 was used for the flowing study as the PRLR ectopically expressed 293 cells. (TIF) [file pone.0252662.s001.tif]

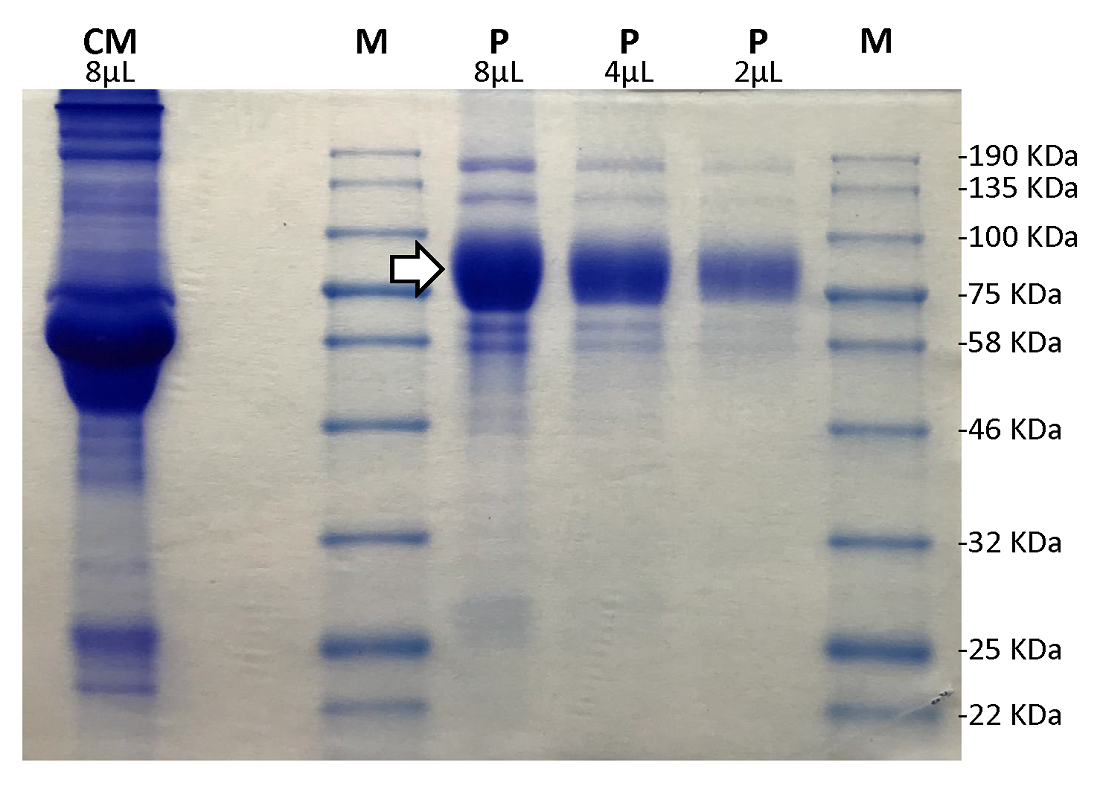

Supplement: S2 Fig — The white arrow indicates the bands of MICA-G129R. CM indicates the lane with the MICA-G129R conditioned media. M indicates the lanes with the protein standard marker. P indicates the lanes with the purified MICA-G129R protein solution. The loading volume of each sample was indicated above each line. (TIF) [file pone.0252662.s002.tif]
