# Supplementary material for: MICA-G129R: A bifunctional fusion protein increases PRLR-positive breast cancer cell death in co-culture with natural killer cells
Source: PLoS One. 2021 Jun 2;16(6):e0252662. doi: 10.1371/journal.pone.0252662 (PMC8172023; doi:10.1371/journal.pone.0252662)

Figure 1B MICA

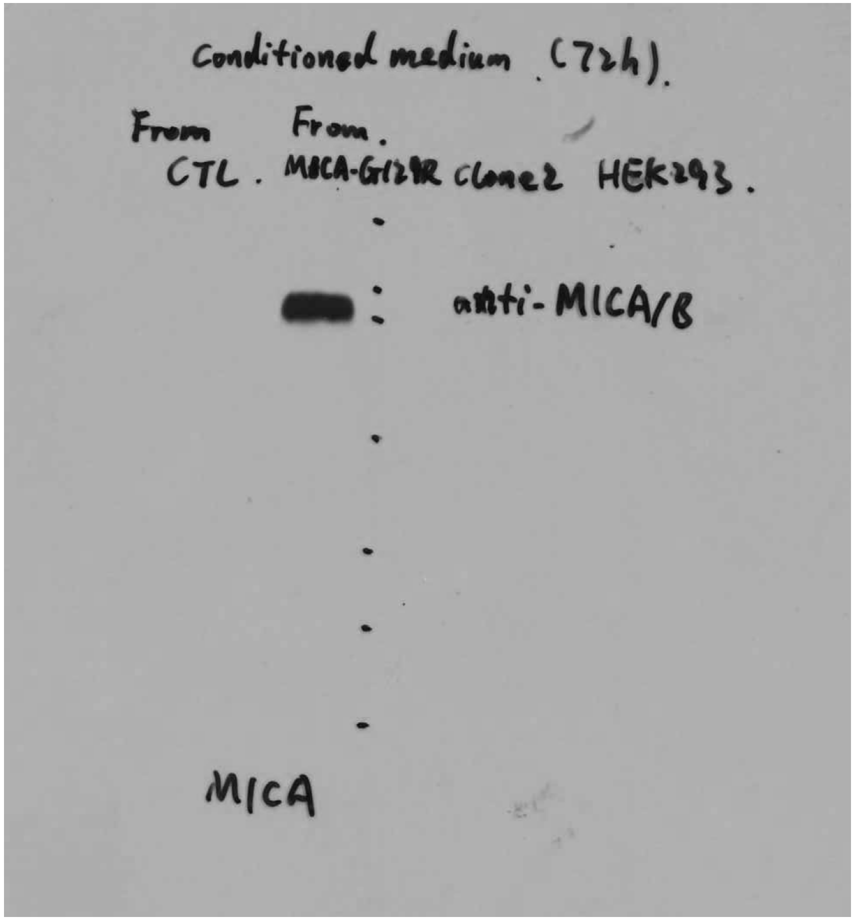

Figure 1B PRLR

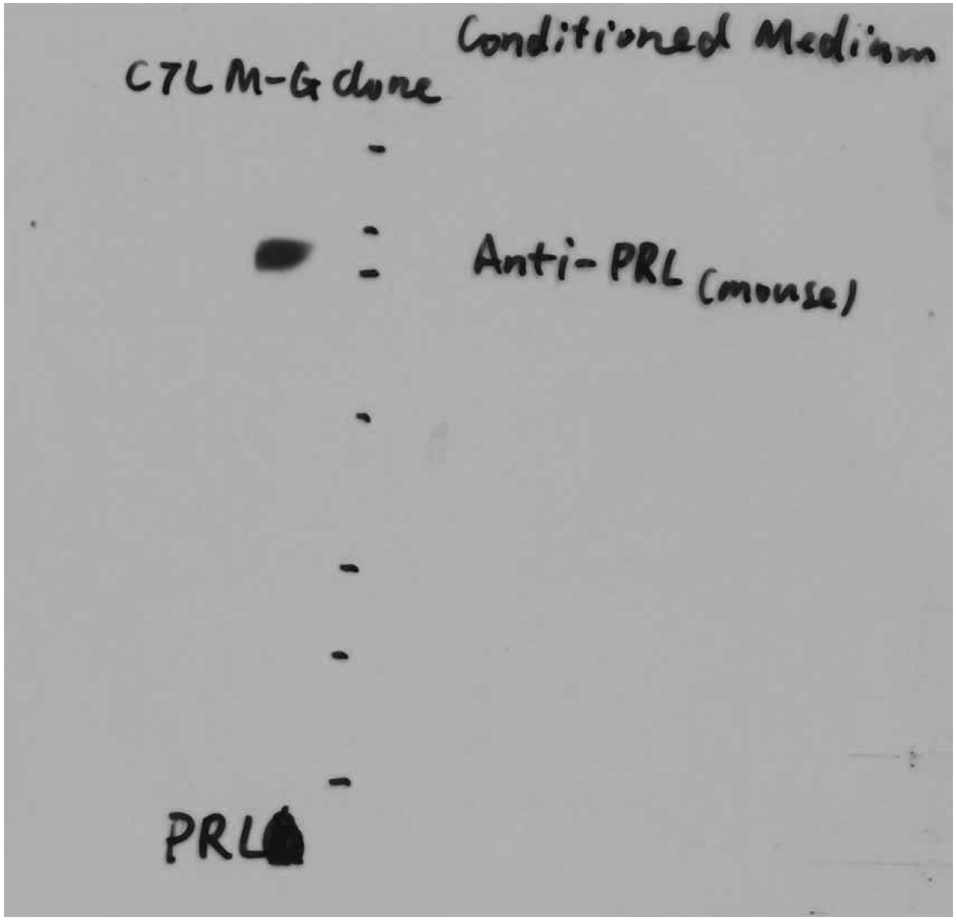

Figure 1B V5

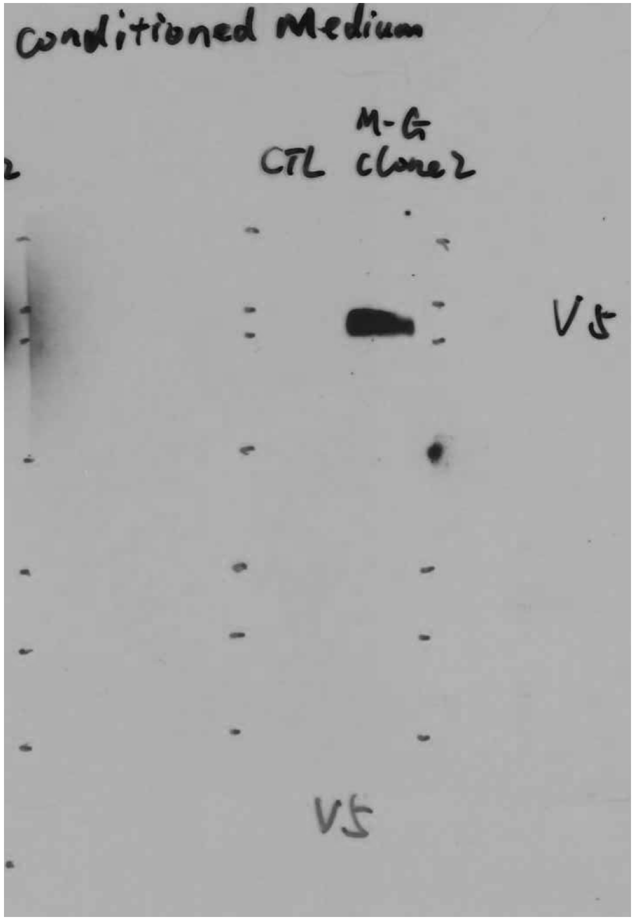

Figure 1C

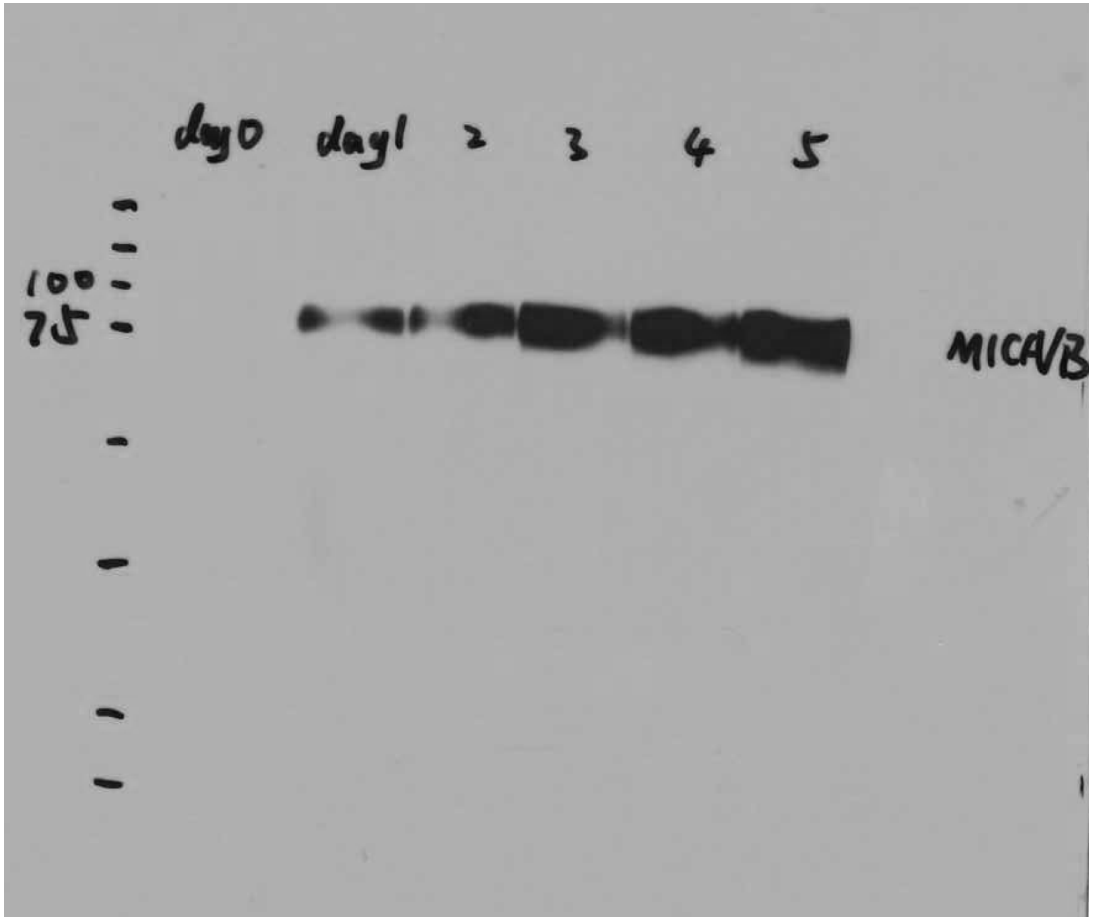

Figure 1E

X
1 2 3

A piece of luminescent tape was used as a marker to indicate the direction of the membrane when developing the film.

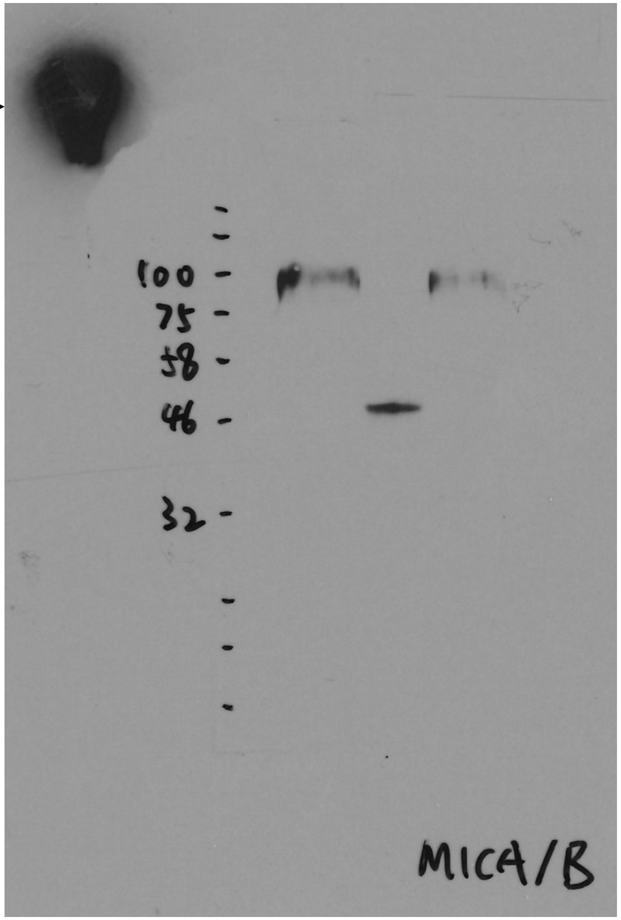

Lane 1: MICA-G129R conditioned medium
Lane 2: MICA conditioned medium
Lane 3: MICA-G129R conditioned medium

Figure 1F

X X X
1 2 3 4 5

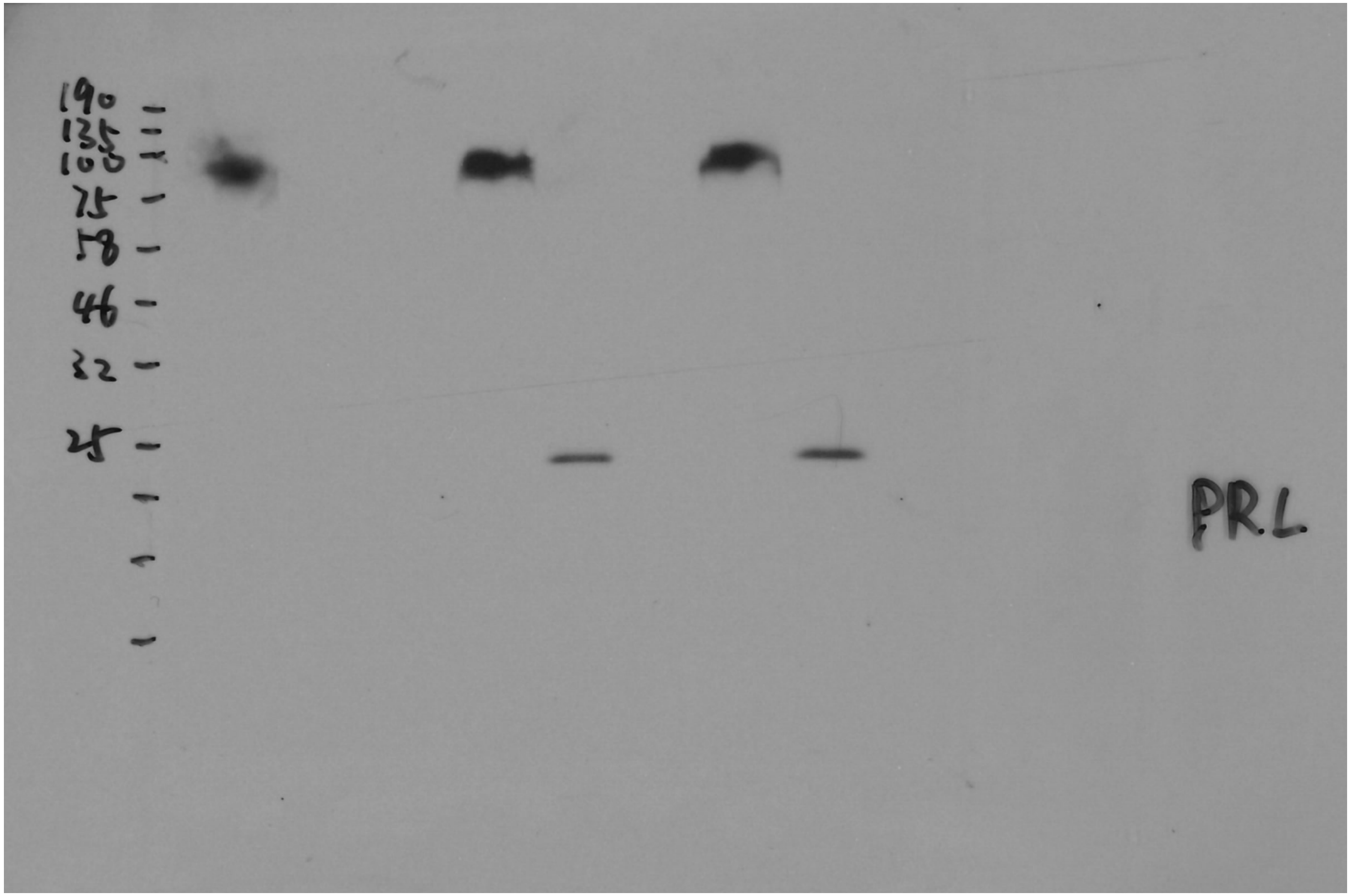

Lane 1: MICA-G129R conditioned medium
Lane 2: G129R conditioned medium
Lane 3: Empty
Lane 4: MICA-G129R conditioned medium
Lane 5: G129R conditioned medium

Figure 2B

A piece of luminescent tape was used as a marker to indicate the direction of the membrane when developing the film.

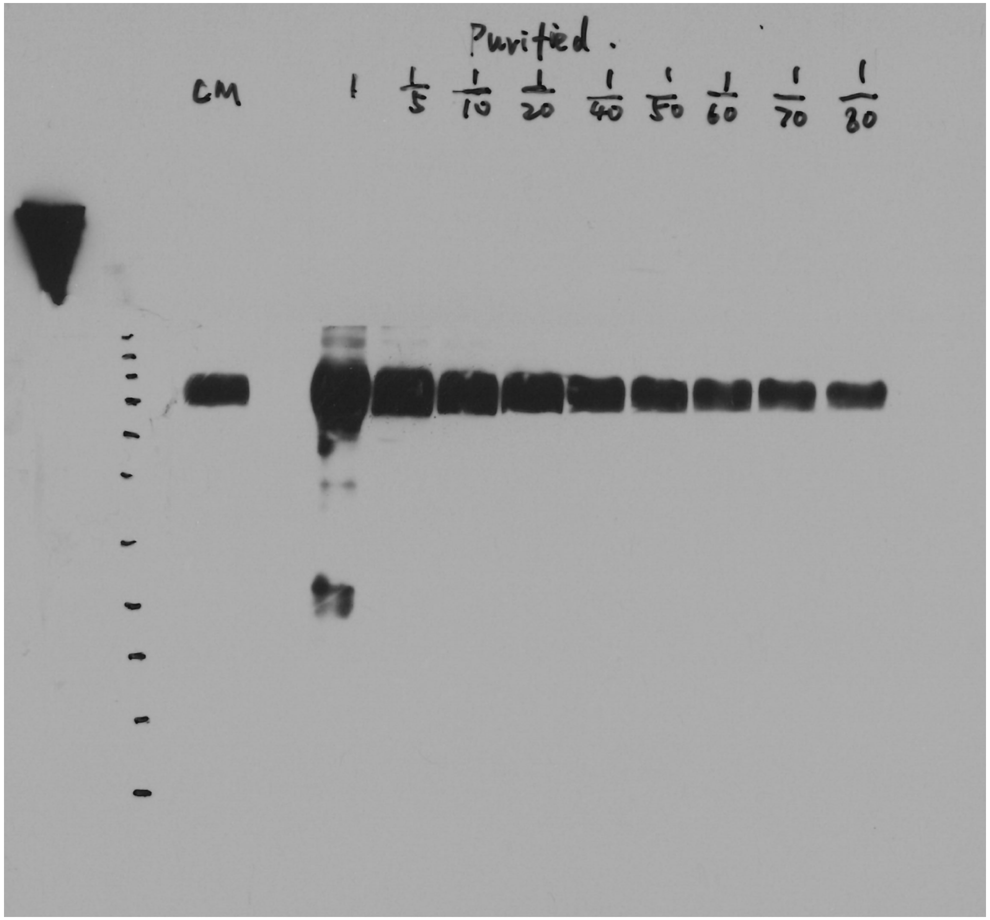

Figure S1 PRLR

X

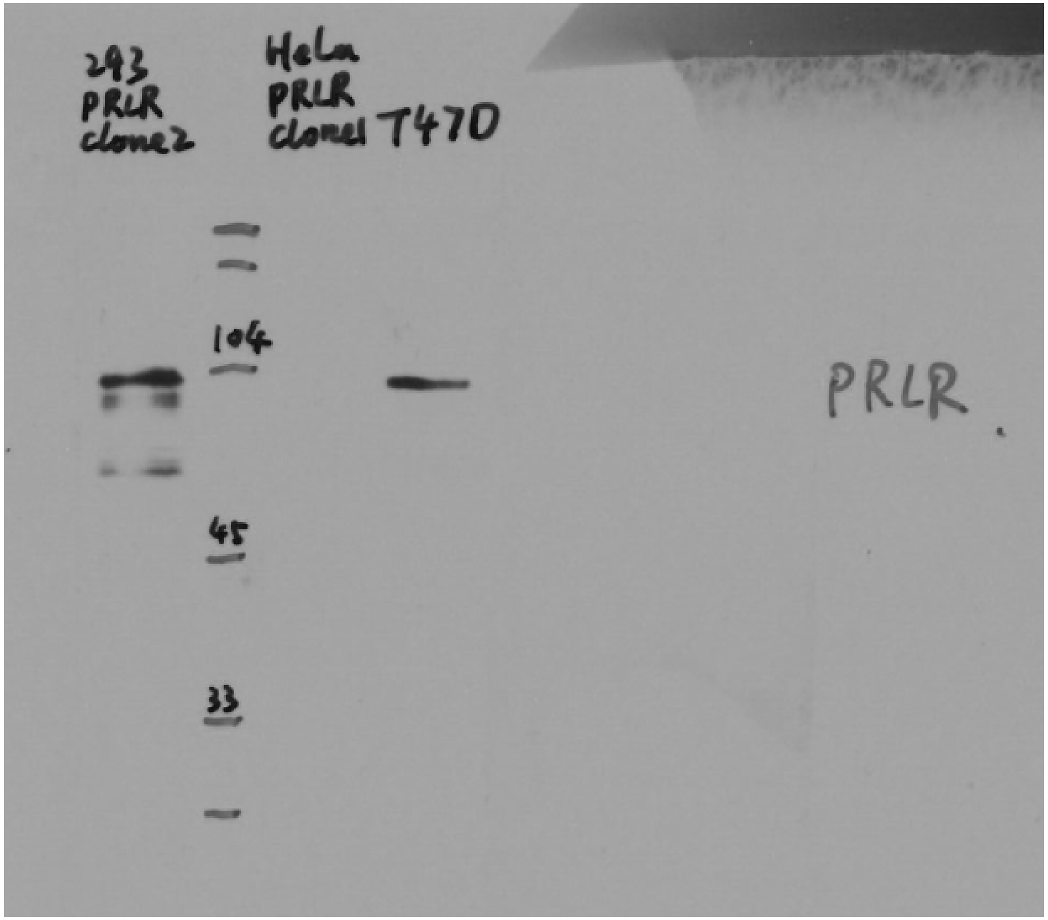

Figure S1 GAPDH

A piece of luminescent tape was used as a marker to indicate the direction of the membrane when developing the film.

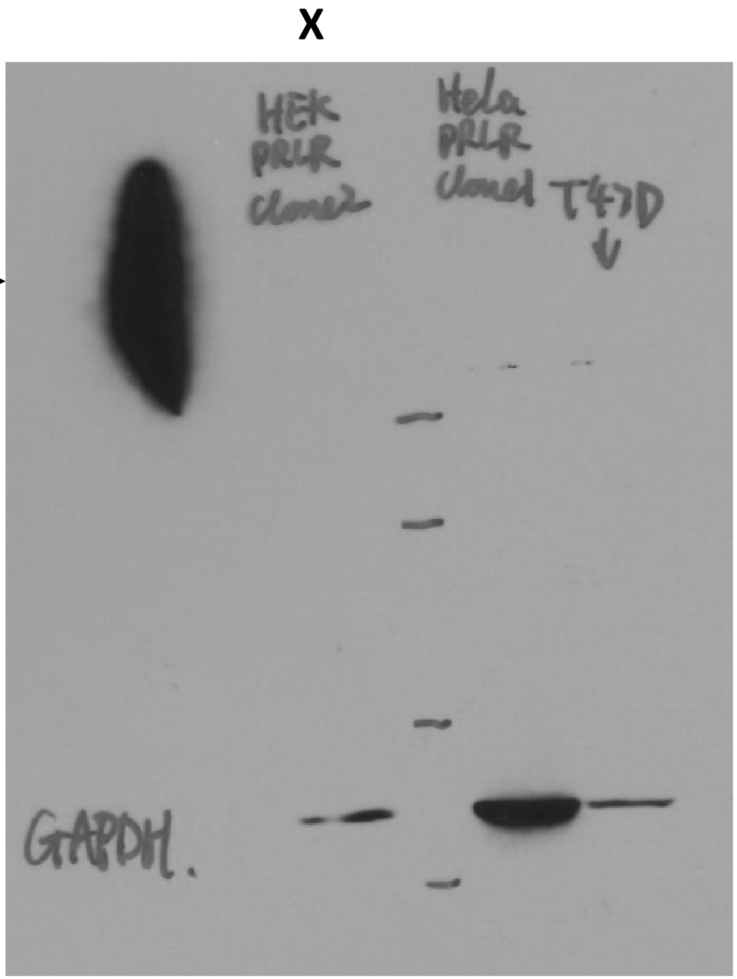

Figure S1B PRLR

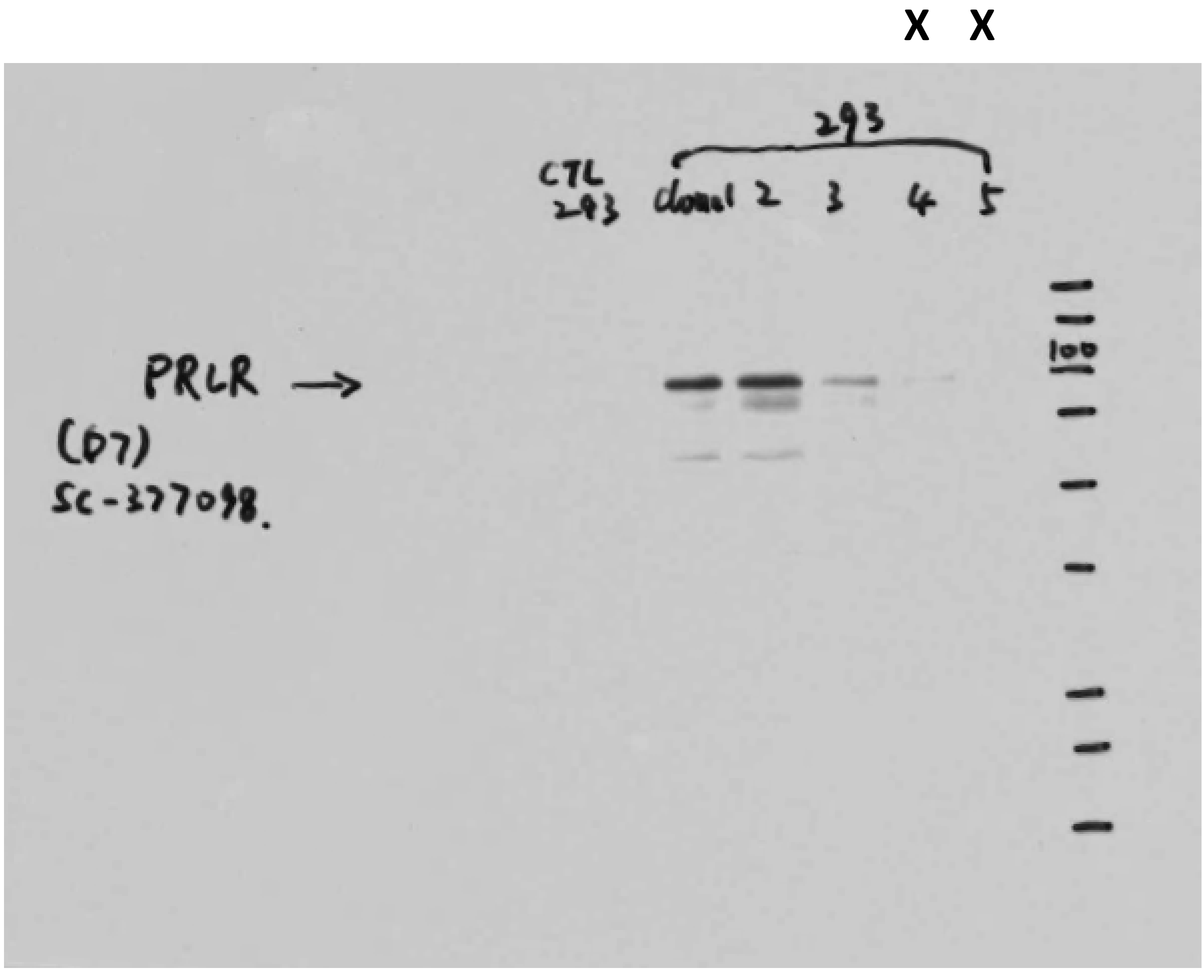

Figure S1B GAPDH

Anti-PRLR antibody was not stripped well.

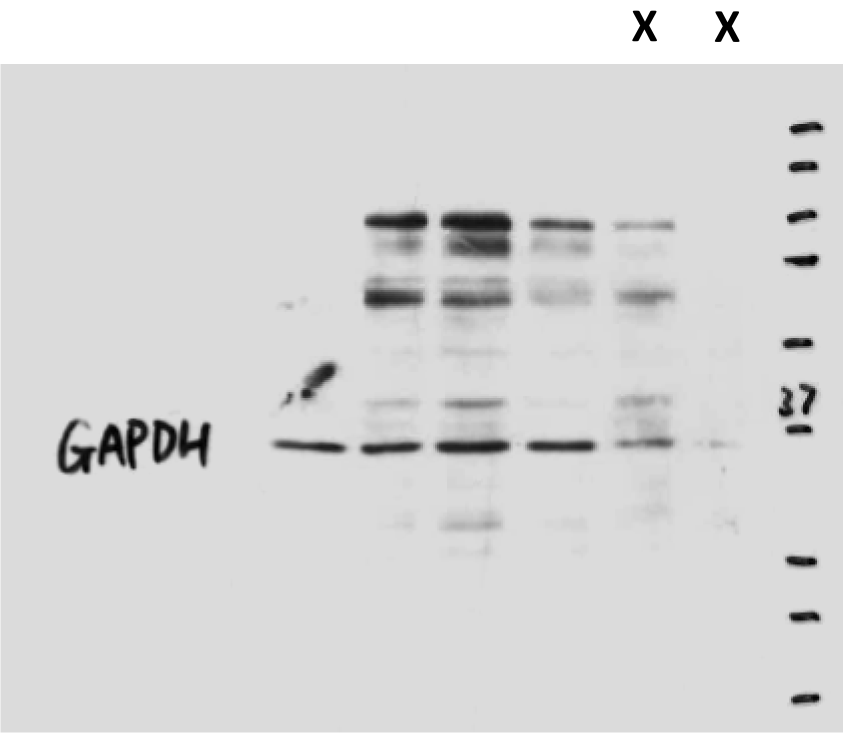

Supplement: S1 Raw images — (PDF) [file pone.0252662.s003.pdf]
